# Blood gas analyzers enable reliable measurement of synovial pH, lactate, and glucose in native and periprosthetic joint infection: an analytical agreement study

**DOI:** 10.5194/jbji-11-247-2026

**Published:** 2026-04-27

**Authors:** Dirk Müller, Igor Lazic, Benjamin Schloßmacher, Vincent Lallinger, Christian Wendler, Rüdiger von Eisenhart-Rothe, Susanne Weber

**Affiliations:** 1 Technical University of Munich, School of Medicine, TUM Klinikum Rechts der Isar, Department of Orthopedic Surgery, Ismaninger Str. 22, 81675 Munich, Germany; 2 Technical University of Munich, School of Medicine, TUM Klinikum Rechts der Isar, Institute of Clinical Chemistry and Pathobiochemistry, Ismaninger Str. 22, 81675 Munich, Germany

## Abstract

**Background:** Synovial pH, lactate, and glucose are established biomarkers for septic arthritis in native joints and have emerging utility in periprosthetic joint infection (PJI). In routine care, these biomarkers are commonly analyzed in central laboratories, which may delay clinical decision-making. Blood gas analyzers (BGAs), which can also measure these parameters, are widely available at the point of care, and their use could accelerate decision-making. However, BGAs are not validated for synovial fluid analysis. **Materials and methods:** This prospective analytical agreement study included 35 consecutive patients undergoing knee joint aspiration for suspected PJI or septic arthritis of the native joint. Each sample was measured in triplicate both in the central laboratory and using a BGA. The agreement between the two methods was assessed using Passing–Bablok and Bland–Altman analyses. The study was designed to assess analytical agreement between both methods rather than diagnostic accuracy. **Results: **BGA measurements for synovial pH, lactate, and glucose demonstrated good to excellent agreement with those obtained using central laboratory methods. Agreement was excellent for synovial glucose and overall good for lactate, with negligible mean bias. Linear regression showed very strong correlations for glucose ( r= 0.997) and lactate ( r= 0.989). Synovial pH showed greater variability, with a mean bias of - 0.10 pH units; however, repeatability analysis revealed lower within-sample variability for BGA-based pH measurements compared with laboratory pH measurements. **Conclusion:** BGAs enable rapid, reliable measurement of synovial pH, lactate, and glucose from small sample volumes and may support timely clinical decision-making in suspected septic arthritis and PJI. Further studies should assess inter-device generalizability and establish device-specific reference ranges.

## 1 Introduction

Periprosthetic joint infection (PJI) is among the most serious complications following total joint arthroplasty, with a reported incidence of approximately 1 %–2 % (Kamath et al., 2015; Nikolaus et al., 2016). As the number of joint arthroplasties continues to rise worldwide, the absolute burden of PJI and the associated need for revision surgery are expected to increase correspondingly, underscoring the importance of accurate and timely diagnosis to guide appropriate treatment strategies (Inacio et al., 2017; Zimmerli et al., 2004).

Synovial fluid analysis represents a cornerstone of contemporary diagnostic algorithms for PJI and is integral to the definitions proposed by the Musculoskeletal Infection Society (MSIS) in 2018, and the European Bone and Joint Infection Society (EBJIS) in 2021 (Parvizi et al., 2018; McNally et al., 2021). Synovial leukocyte count and the percentage of polymorphonuclear neutrophils (PMN%) remain essential biomarkers in the diagnostic work-up of suspected PJI and are widely used in clinical practice (Sabater-Martos et al., 2024; Zmistowski et al., 2012).

Synovial pH, lactate, and glucose concentrations are well-established parameters in the diagnosis of native joint septic arthritis (Shirtliff Mark and Mader Jon, 2002; Gobelet and Gerster, 1984; Lenski and Scherer, 2014b; Carpenter et al., 2011; Riley, 1981; Brennan and Hsu, 2012; Elsissy et al., 2020; Söderquist, 1998). In line with this evidence, the 2023 EBJIS guideline for septic arthritis in the native joint (SANJO) recommends the analysis of synovial lactate and glucose as part of the diagnostic work-up (Ravn et al., 2023). More recently, the diagnostic utility of synovial pH, lactate, and glucose in the context of PJI was also investigated (Theil et al., 2022; Judl et al., 2024; Sabater-Martos et al., 2025; Müller et al., 2025). In a previous study, we demonstrated that the combined assessment of synovial pH, lactate, and glucose achieved a high negative predictive value of 84 % for ruling out PJI (Müller et al., 2025). Notably, the serum-to-synovial glucose ratio showed a sensitivity of 72 % and a specificity of 94 % for the diagnosis of acute PJI (Sabater-Martos et al., 2025).

In routine clinical practice, synovial pH, lactate, and glucose are typically measured in the central laboratory of hospitals using large analytical platforms, which may be associated with delays in result availability. Moreover, synovial leukocyte count and PMN% are not consistently accessible in all institutions, particularly outside routine laboratory hours. Blood gas analyzers (BGAs), which are widely available as point-of-care (POC) devices in emergency departments and intensive care units, are also able to measure pH, lactate, and glucose. Although BGAs are currently not validated for the analysis of synovial fluid, previous studies have successfully used BGAs to measure synovial pH (Theil et al., 2022).

Accordingly, the purpose of this study was to evaluate the feasibility of BGA-based measurements of synovial pH, lactate, and glucose, as well as their analytical agreement with central laboratory methods.

## 2 Materials and methods

### 2.1 Study design and patient selection

This prospective study included consecutive patients undergoing knee joint aspiration for suspected PJI of a knee arthroplasty or suspected septic arthritis of the native knee joint. Both infected and non-infected native joints and knee arthroplasties were included to ensure a broad range of analyte concentrations and synovial fluid viscosities. All joint aspirations were performed under sterile conditions in the outpatient department. The knee joint was selected as the study focus because sufficient synovial fluid volume was required for multiple analyses, and knee effusions typically provide adequate sample volumes. Patients from whom less than 5 mL of synovial fluid could be obtained were excluded ( n= 10). A total of 35 patients were included in the study, comprising 10 native knee joints and 25 knee arthroplasties.

### 2.2 Ethics

Ethical approval was waived by the Ethics Committee of the Technical University of Munich (reference number 714/20S, 20 November 2020). Written informed consent was obtained from all participants prior to inclusion. This study was conducted and reported in accordance with the STARD 2015 guidelines for diagnostic accuracy studies.

### 2.3 Synovial fluid analysis

**Figure 1:**
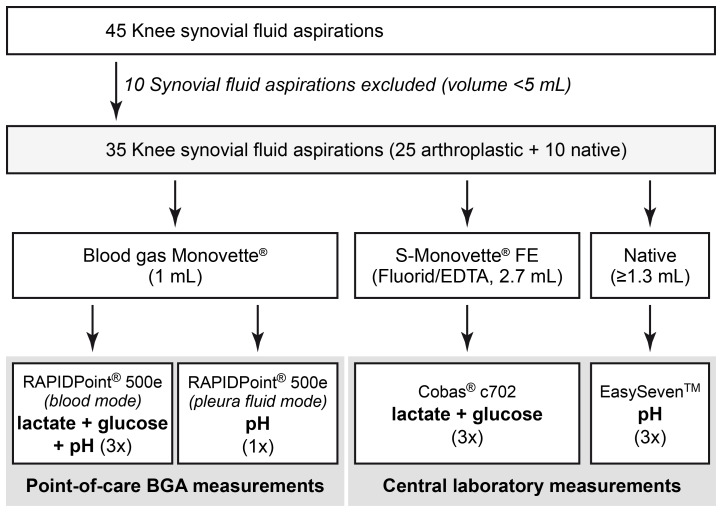
Flowchart of synovial fluid analysis workflow.

All synovial fluid samples were processed immediately after aspiration. Aspirated synovial fluid was transferred to Blood Gas Monovette^®^ and S-Monovette^®^ Fluoride/EDTA FE (both Sarstedt, Nümbrecht, Germany) collection tubes for BGA analysis at the POC and analysis of lactate or glucose in the central laboratory, respectively. Native synovial fluid was used for pH measurements in the central laboratory. In the laboratory setting, synovial pH was measured using a calibrated SevenEasy^™^ pH analyzer (Mettler-Toledo, Columbus, OH, USA) at room temperature (23 °C), while synovial lactate and synovial glucose concentrations were determined using the central laboratories Cobas^®^ c702 clinical chemistry analyzer (Roche Diagnostics, Basel, Switzerland). POC BGA measurements were performed using a RAPIDPoint^®^ 500e analyzer (Siemens Healthineers, Erlangen, Germany). Each prepared sample was measured three times, avoiding relevant time delays between the applied methods. In addition, one synovial pH measurement per sample was performed, using the BGA set to pleural fluid mode at room temperature (23 °C). The workflow of synovial fluid analysis is illustrated in Fig. 1.

### 2.4 Statistical analysis

For statistical analyses, the mean value derived from three repeated measurements per sample was used, which reduced random analytical variability. Agreement between BGA and central laboratory measurements for synovial pH, lactate, and glucose was assessed using Passing–Bablok and Bland–Altman analysis. Results outside the analytical range (Table 3) were excluded from statistical agreement analyses, as these values could not be reliably quantified. The mean difference (bias) and 95 % limits of agreement (mean ± 1.96 standard deviations) were calculated and graphically displayed. In addition, linear Deming regression analysis was performed to evaluate the relationship between BGA and central laboratory results, and the 95 % confidence interval (CI) for regression parameters (i.e., slope and intercept) as well as results from the cumulative sum (CUSUM) test of residuals were reported. The assumption of normally distributed residuals was evaluated through graphical inspection of the distribution of differences between methods, including histograms and normal P–P plots of regression-standardized residuals, as well as by applying the Shapiro–Wilk test. Homoscedasticity of residuals was assessed by testing for heteroscedasticity using the Breusch–Pagan test and by visually examining Bland–Altman plots for potential trends in variability of differences across the measurement range. In order to complete the description of the association strength between both methods, Pearson's correlation coefficients ( r ) were calculated.

Precision was evaluated from triplicate measurements for each sample and method. For synovial lactate and glucose, precision was expressed as coefficients of variation (CVs), whereas precision of synovial pH was expressed as standard deviation (SD) in pH units.

All analyses were performed using SPSS Statistics (version 29; IBM Corp., Armonk, NY, USA) and OriginPro (version 2025; OriginLab Corporation, Northampton, MA, USA). CUSUM testing was performed with the R “strucchange” package. A two-sided p value ≤ 0.05 was considered statistically significant where applicable.

## 3 Results

### 3.1 Overall analyzable results

In total, synovial fluid samples from 35 patients were collected and analyzed. Four samples yielded synovial pH values outside the quantifiable range of the BGA (Table 3), and an additional three samples did not provide reliable pH measurements using the central laboratory pH meter due to insufficient remaining sample volumes. For synovial lactate, one sample exceeded the analytical range of the Cobas^®^ c702 analyzer at the central laboratory and was not further diluted for analysis, while another sample could not be analyzed with the Cobas^®^ c702 analyzer because of excessive viscosity, despite hyaluronidase treatment. Furthermore, five and four samples were below the lower limit of quantification for glucose using the BGA and central laboratory device, respectively. In all cases where BGA analyses failed to yield definitive results due to values falling outside the device's measurement ranges, the findings were consistent with those obtained from central laboratory measurements. Consequently, in-depth comparative analyses between BGA and central laboratory measurements were performed on 29 samples for pH, on 33 for lactate, and on 30 for glucose, as detailed in the following sections. All analyses indicated normally distributed residuals. Furthermore, the results of the Breusch–Pagan tests, supported by a visual inspection of Bland–Altman plots, revealed no evidence of systematic heteroscedasticity across the measurement range for the analyzed biomarkers.

### 3.2 Synovial pH measurements

**Figure 2:**
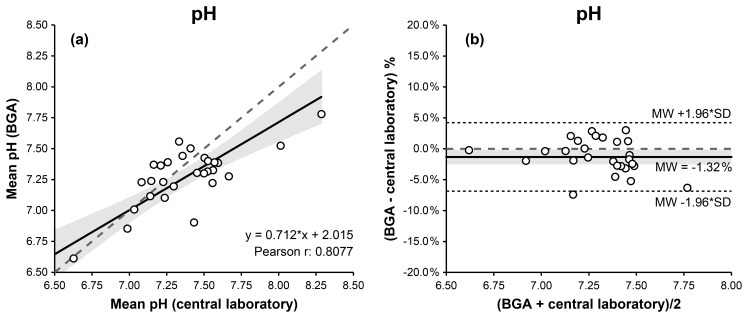
Comparison of BGA and central laboratory pH measurements in blood mode using Passing–Bablok **(a)** and Bland–Altman **(b)** plots. The Passing–Bablok plot shows mean BGA pH versus mean central laboratory pH values, with the line of identity (dashed) and the linear Deming regression (solid). The Bland–Altman plot displays % differences (mean BGA pH – central laboratory pH) against the mean pH of both measurements, with the mean bias (solid), the 95 % limits of agreement (dotted), and the 0 % bias (dashed). The 95 % confidence intervals for the Deming regression line and the mean bias are highlighted in gray.

For synovial pH measurements in blood mode, Passing–Bablok and Deming regression analyses demonstrated a linear relationship between BGA and laboratory measurements. Pearson's correlation coefficient indicated a statistically significant association ( r= 0.808; p<0.001 ). The relationship was described by the following Deming regression equation: BGA pH = 0.712 × laboratory pH + 2.015 (Fig. 2a, Table 1).

The CUSUM test showed no significant deviation from linearity ( S = 0.796, p = 0.142). Bland–Altman analysis indicated good agreement, with a mean bias of - 0.10 pH units and 95 % limits of agreement ranging from - 0.51 to + 0.31 pH units (Fig. 2b, Table 1).

With the BGA set to pleural fluid mode at 23 °C, Bland–Altman analysis indicated better agreement between BGA and laboratory pH measurements, yielding a mean bias of - 0.04 pH units with 95 % limits of agreement ranging from - 0.37 to + 0.28 pH units. However, due to the narrower analytical range of the pleural fluid mode compared with the conventional BGA pH mode, only 22 (62 %) result pairs were eligible for statistical analysis.

### 3.3 Synovial lactate measurements

**Figure 3:**
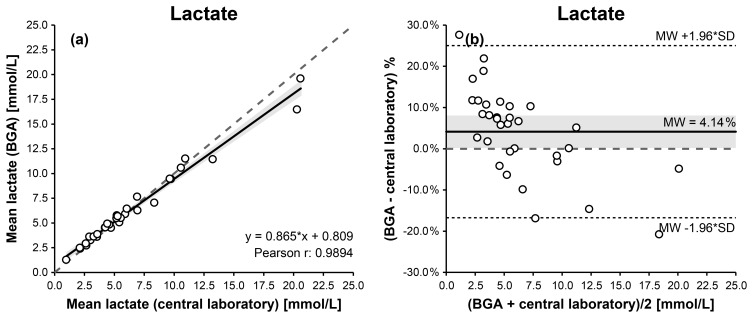
Comparison of BGA and central laboratory lactate measurements using Passing–Bablok **(a)** and Bland–Altman **(b)** plots. The Passing–Bablok plot shows mean BGA lactate versus mean central laboratory lactate concentrations with the line of identity (dashed) and the linear Deming regression (solid). The Bland–Altman plot displays % differences (mean BGA lactate – mean central laboratory lactate) against the mean lactate concentration of both measurements, with the mean bias (solid), the 95 % limits of agreement (dotted), and the 0 % bias (dashed). The 95 % confidence intervals for the Deming regression line and the mean bias are highlighted in gray.

For synovial lactate measurements, Passing–Bablok and Deming regression analyses demonstrated a linear relationship between BGA and central laboratory results. Pearson's correlation coefficient indicated a statistically significant association ( r= 0.989; p<0.001 ). The relationship was described by the following Deming regression equation: BGA lactate = 0.865 × laboratory lactate + 0.809 (Fig. 3a, Table 1).

Linearity between methods was confirmed by the CUSUM test ( S = 0.664, p = 0.302). Bland–Altman analysis indicated good overall agreement, with a mean bias of - 0.04 mmol L^−1^ and 95 % limits of agreement ranging from - 1.77 to + 1.69 mmol L^−1^ (Fig. 3b, Table 1), with some dispersion observed at lower and higher concentration ranges.

### 3.4 Synovial glucose measurements

**Figure 4:**
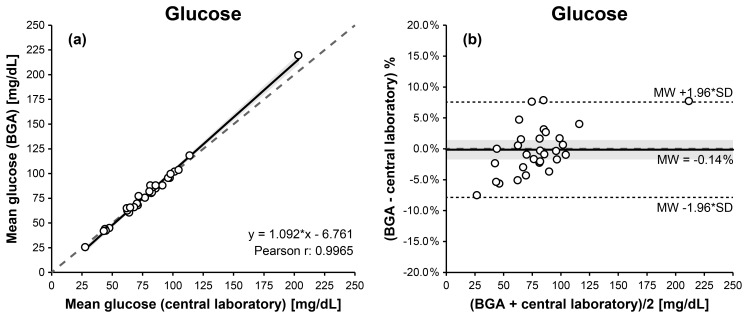
Comparison of BGA and central laboratory glucose measurements using Passing–Bablok **(a)** and Bland–Altman **(b)** plots. The Passing–Bablok plot shows mean BGA glucose versus mean central laboratory glucose concentrations with the line of identity (dashed) and the linear Deming regression (solid). The Bland–Altman plot displays % differences (mean BGA glucose – mean central laboratory glucose) against the mean glucose concentration of both measurements, with the mean bias (solid), the 95 % limits of agreement (dotted), and the 0 % bias (dashed). The 95 % confidence intervals for the Deming regression line and the mean bias are highlighted in gray.

For synovial glucose measurements, Passing–Bablok and Deming regression analyses demonstrated a linear relationship between BGA and central laboratory results. Pearson's correlation coefficient indicated the strongest statistically significant association among the three biomarkers ( r= 0.997; p<0.001 ). The relationship was described by the following Deming regression equation: BGA glucose = 1.092 × laboratory glucose - 6.761 (Fig. 4a, Table 1).

A mild proportional bias was observed at higher glucose concentrations, without relevant clinical impact. Linearity between methods was confirmed by the CUSUM test ( S = 0.786, p = 0.151). Bland–Altman analysis indicated excellent agreement, with a mean bias of + 0.58 mg dL^−1^ and 95 % limits of agreement ranging from - 7.11 to + 8.27 mg dL^−1^ (Fig. 4b, Table 1).

**Table 1:** Agreement and correlation between blood gas analyzer (BGA) and central laboratory measurements. Bias and 95 % limits of agreement (LoA) were derived from Bland–Altman analysis. Slope and intercept parameters were obtained from linear Deming regression analysis of the Passing–Bablok plot; and 95 % confidence intervals (CIs) of calculated bias and regression parameters are given in parentheses. Deviation from linearity was assessed by CUSUM testing. Pearson correlation coefficients ( r ) are reported for descriptive purposes.

Analyte	Bland–Altman analysis		Passing–Bablok and Deming regression analysis			
	Bias (95 % CI)	95 % LoA	Slope (95 % CI)	Intercept (95 % CI)	CUSUM p value	r
Synovial pH (blood mode)	- 0.10 pH units ( - 0.18 to - 0.02)	- 0.51 to + 0.31 pH units	0.712 (0.50–0.93)	2.015 pH units ( + 0.43 to + 3.60)	0.14	0.808
Synovial pH (pleural fluid mode)	- 0.04 pH units ( - 0.11 to + 0.03)	- 0.37 to + 0.28 pH units	0.344 (0.02–0.67)	4.786 pH units ( + 2.41 to + 7.16)	0.43	0.470
Synovial lactate	- 0.04 mmol L^−1^ ( - 0.35 to + 0.27)	- 1.77 to + 1.69 mmol L^−1^	0.87 (0.82–0.91)	0.81 mmol L^−1^ ( + 0.45 to + 1.17)	0.30	0.989
Synovial glucose	+0.58 mg dL^−1^ ( - 0.88 to + 2.05)	-7.11 to + 8.27 mg dL^−1^	1.09 (1.06–1.13)	- 6.76 mg dL^−1^ ( - 9.78 to - 3.74)	0.15	0.997

### 3.5 Precision of measurements

In order to assess the precision of BGA analyses, within-run variability derived from triplicate measurements was evaluated. Precision analysis based on triplicate measurements demonstrated slightly lower variability in BGA measurements compared with central laboratory measurements for synovial pH. For this analyte, precision was expressed as the standard deviation (SD) in pH units, reflecting the logarithmic scale of pH. BGA measurements showed a mean SD of 0.024 ± 0.021 pH units, compared with 0.030 ± 0.028 pH units for central laboratory measurements. In contrast, central laboratory measurements exhibited lower variability for synovial lactate and glucose when expressed as coefficients of variation (CVs) (Table 2).

**Table 2:** Precision of blood gas analyzer (BGA) and central laboratory measurements. Precision was derived from triplicate measurements and is expressed as standard deviation (SD) in pH units for synovial pH and as coefficients of variation (CVs) for synovial lactate and glucose. All measurements showed low variability, indicating good analytical precision. SD: standard deviation; CV: coefficient of variation; n : number of samples included in the precision analysis.

Analyte	Method	n	SD range	Median SD	Mean SD ± SD
			(pH units)	(pH units)	(pH units)
Synovial pH	BGA	31	0.002–0.095	0.016	0.024 ± 0.021
	Central laboratory	32	0.006–0.133	0.020	0.030 ± 0.028
Analyte	Method	n	CV range	Median CV	Mean CV ± SD
			(%)	(%)	(%)
Synovial lactate	BGA	35	0.52–9.36	1.76	2.58 ± 2.24
	Central laboratory	33	0.00–5.97	0.57	2.24 ± 1.50
Synovial glucose	BGA	30	0.00–3.01	0.71	0.88 ± 0.80
	Central laboratory	31	0.00–2.55	0.71	0.85 ± 0.65

## 4 Discussion

The principal finding of this study is that BGAs can reliably measure synovial pH, lactate, and glucose with good analytical agreement with central laboratory methods. By enabling near-patient testing, the use of BGAs for synovial fluid analysis may accelerate the availability of results in the diagnostic workflow and represents a reliable, rapid, and cost-effective approach for the measurement of these biomarkers in suspected septic arthritis and periprosthetic joint infection, without reliance on more complex or time-consuming laboratory analyses.

### 4.1 Synovial pH measurements

Previous studies have demonstrated favorable diagnostic performance of synovial pH in the diagnosis of PJI, with reported sensitivities ranging from 50 % to 89 % and specificities between 81 % and 96 % when cut-offs between 7.11 and 7.40 were used (Theil et al., 2022; Judl et al., 2024; Müller et al., 2025).

Among the parameters analyzed in this study, synovial pH proved to be the most challenging to measure. It showed the greatest variability between different measurement methods. pH is a highly sensitive parameter that is influenced by multiple physiological and pre-analytical factors, particularly temperature fluctuations (Cheng et al., 1998; Ashwood et al., 1983; Mishra and Rahman, 2009). In addition, delays between sample collection and analysis can lead to artificially increased pH values due to a reduction in p CO_2_ caused by carbon dioxide diffusion into ambient air (Cheng et al., 1998; Venkatesh et al., 1999; Mishra and Rahman, 2009). For this reason, as with conventional blood gas analysis with blood samples, synovial fluid blood gas analysis should ideally be performed immediately after joint aspiration whenever feasible.

The determination of pleural fluid pH provides a useful analogy. Pleural pH is an established diagnostic parameter in the evaluation of pleural effusions, with pH values below 7.20 indicating pleural empyema requiring tube thoracostomy (Houston, 1981; Good et al., 1980). Historically, pleural fluid pH has been measured using indicator strips, pH meters, or BGAs. Comparative studies have consistently demonstrated the superiority of BGAs over pH meters, showing that only BGA-derived pH values achieve sufficient accuracy for clinical decision-making (Cheng et al., 1998; Venkatesh et al., 1999; Mishra and Rahman, 2009; Lesho and Roth, 1997). The superiority of BGA pH measurements over pH meter analyses was also observed in this study, by showing less variability in repeated measurements and more reliable results.

Despite this evidence, approximately 30 %–50 % of laboratories in the United States reportedly continue to use inaccurate methods for pleural fluid pH measurement (Putnam et al., 2013; Bowling et al., 2012). This observation underscores the importance of standardized measurement techniques and supports the use of BGAs for synovial pH assessment in the diagnostic work-up of PJI and SANJO.

### 4.2 Synovial lactate measurements

Elevated synovial lactate concentrations are indicative of joint infection. Previous studies investigating PJI have reported sensitivities ranging from 50 % to 73 % and specificities between 67 % and 88 %, using cut-off values between 5.30 and 8.45 mmol L^−1^ (Sharma et al., 2020; Lenski and Scherer, 2014a, 2015; Müller et al., 2025). In addition, the 2023 EBJIS guideline on SANJO recommends the analysis of synovial lactate as part of the diagnostic work-up (Ravn et al., 2023).

In the present study, synovial lactate concentrations measured using the BGA showed good agreement with central laboratory measurements, with negligible mean bias.

Comparable findings have been reported in other clinical contexts. A recent study evaluating cerebrospinal fluid lactate determination for the diagnosis of acute meningitis found a small and systematic difference between results obtained with a RAPIDPoint 500 BGA and conventional laboratory assays (Fernández Reina et al., 2024). Despite this bias, the authors concluded that BGA-based lactate quantification remains a useful diagnostic tool, supporting the applicability of this approach for synovial fluid analysis.

### 4.3 Synovial glucose measurements

Low synovial glucose concentrations are associated with septic arthritis in native joints (Söderquist, 1998) as well as PJI (Lenski and Scherer, 2014a, 2015; De Vecchi et al., 2016; Haertlé et al., 2022; Sabater-Martos et al., 2025). The 2023 EBJIS guideline on SANJO suggests the analysis of synovial glucose as an additional adjunct investigation (Ravn et al., 2023).

Because synovial glucose concentrations may be influenced by fluctuations in blood glucose levels (Brennan and Hsu, 2012), Sabater-Martos et al. proposed the serum : synovial glucose ratio as a diagnostic parameter for acute PJI. Using a cut-off value of > 0.69, they reported a sensitivity of 72 % and a specificity of 94 % (Sabater-Martos et al., 2025).

In our comparative analysis, synovial glucose concentrations obtained by using BGAs showed excellent agreement with central-laboratory-based glucose measurements, revealing only minimal variability between methods.

Similar observations have been reported for pleural fluid glucose measurements. Based on the robustness and reproducibility of glucose analysis, Mishra et al. (2009) suggested that pleural glucose levels may be used to support clinical decision-making when accurate pleural pH measurements are not available (Mishra and Rahman, 2009). These findings further support the reliability of synovial glucose results obtained as an adjunct diagnostic parameter using BGAs.

### 4.4 Measurement range of the RAPIDPoint 500e BGA

Some BGAs, such as the Siemens RAPIDPoint 500e, offer a dedicated mode for pleural fluid measurement. In our study, measurements performed in pleural fluid mode, calibrated to 23 °C, showed closer agreement with pH meter values. However, the pleural fluid mode is limited by a narrower analytical range of pH 7.000–7.500 (Table 3), allowing statistical analysis of only a limited set of samples.

**Table 3:** Analytical measurement range of the Siemens RAPIDPoint 500e BGA (RAPIDPoint 500e System Operator's Guide 10631336 Rev. F, 2025-05).

Analyte	Units	Reporting range	Resolution
pH	–	6.500–7.800	0.001
Pleural pH	–	7.000–7.500	0.001
Lactate	mmol L^−1^	0.18–30.00	0.01
Glucose	mg dL^−1^	20–750	1

This limitation is of minor clinical relevance for the diagnosis of PJI, as synovial fluid pH values below 7.000 are strongly associated with infection (Müller et al., 2025; Theil et al., 2022; Judl et al., 2024). Consequently, values below the quantification range are, in practice, already highly suggestive of PJI.

Similar considerations apply to the measurement ranges of synovial lactate and glucose. Extremely low synovial glucose concentrations ( < 20 mg dL^−1^) or markedly elevated synovial lactate levels ( > 30 mmol L^−1^) are strongly associated with infection (Müller et al., 2025). Therefore, even when values approach or exceed the upper or lower quantification limits of the BGA, their diagnostic interpretation remains clinically meaningful.

### 4.5 Possible damage to BGAs and future directions

Synovial fluid exhibits a substantially higher viscosity than blood (Roškar et al., 2025; Fu et al., 2019), raising concerns that its application to BGAs could lead to clogging or damage. In the present study, encompassing 140 synovial fluid measurements, including those with highly viscous samples, no clotting events or BGA malfunctions were observed. Furthermore, consultation with one of the largest German manufacturers of BGAs confirmed that damage to the analyzer itself is highly unlikely. In a worst-case scenario, only the disposable measurement cartridge may be affected.

Comparable concerns were previously raised regarding pleural fluid pH analysis (Cheng et al., 1998); however, pleural fluid measurements using BGAs are now well established and manufacturer approved. Similarly, the development of a dedicated synovial fluid measurement mode by BGA manufacturers could further support standardized and optimized clinical applications.

An important advantage of BGA analysis over conventional laboratory testing is the minimal sample volume required, the feasibility of POC testing, and the rapid turnaround time. While laboratory-based analyses, covering pH electrode measurements and clinical chemical analyses, typically require several milliliters of synovial fluid, BGAs generally require approximately 100 µL . Moreover, due to the high viscosity of most synovial fluid samples, at least in central laboratories equipped with Cobas^®^ Pro c702 analyzers, pre-analytical hyaluronidase treatment of samples is necessary to avoid clogging of the analytical system. Besides significantly prolonging analysis times, this can falsify particularly synovial glucose and lactate concentrations in infected samples and explain some result variations between BGA and central laboratory measurements in the present study. Positioning BGAs at POC eliminates the need for sample transport and processing, thereby accelerating clinical decision-making.

In addition to pH, lactate, and glucose, BGAs provide multiple further diagnostic parameters, including sodium, potassium, partial pressure of carbon dioxide ( p CO_2_), ionized calcium, total hemoglobin, partial pressure of oxygen ( p O_2_), chloride, and CO-oximetry parameters. Notably, p CO_2_ may be of particular interest, as it is already used in the diagnostic evaluation of pleural effusions (Light et al., 1973). Further studies are needed to determine the diagnostic relevance of these parameters in the diagnosis of PJI.

### 4.6 Limitations

The present study has several limitations that are acceptable in the context of this feasibility study. First, only a single BGA model was evaluated; therefore, the findings may not be generalizable to BGAs from other manufacturers, which may yield different results. Although no device damage was observed during the study period, the long-term effects of synovial fluid analysis on BGAs remain unknown, and off-label use may void manufacturer warranties. In addition, BGAs have a limited analytical measurement range (Table 3). Although values exceeding this range are already strongly suggestive of joint infection, excluding measurements outside the analytical range may have introduced bias into the agreement estimates and represents a limitation of the analysis. Finally, agreement analyses were based on the mean of triplicate measurements per sample. While this approach reduces random analytical variability, it may slightly narrow the calculated limits of agreement compared with single measurements typically obtained in routine clinical practice. Treating triplicate measurements as independent observations in the agreement analyses yielded no substantial differences compared with analysis of triplicate means (data not shown), thereby supporting the robustness of our findings and their applicability to clinical routine. Triplicate measurements were also used to assess the precision of the BGA and central laboratory results with synovial fluid samples. Here, it needs to be noted that only within-run or intra-day variabilities were determined, but no inter-run or inter-day variabilities. The limitations outlined, which are beyond the scope of this feasibility study, should be addressed in future performance evaluation studies.

### 4.7 Conclusion

In conclusion, measurement of synovial pH, lactate, and glucose using BGAs demonstrates good analytical agreement with central laboratory methods and represents a reliable, rapid, and cost-effective approach for the assessment of these biomarkers that may support clinical decision-making in suspected septic arthritis and periprosthetic joint infection. These POC analyzers have the potential to facilitate efficient decision-making in routine orthopedic practice. Further studies are warranted to evaluate the performance of BGAs from different manufacturers and to establish brand-specific reference ranges. The development of a dedicated synovial fluid mode by BGA manufacturers would be highly desirable to facilitate standardized and optimized clinical use.

## Data Availability

The datasets used and/or analyzed during the current study are available from the corresponding author on reasonable request.

## Appendix A Abbreviations

**Table:**

BGA	Blood gas analyzer
CV	Coefficient of variation
EBJIS	European Bone and Joint Infection Society
ICM	International consensus meeting
MSIS	Musculoskeletal Infection Society
PJI	Periprosthetic joint infection
PMN%	Polymorphonuclear percentage
POC	Point of care
SANJO	Septic arthritis in native joints
STARD	Standards for reporting of diagnostic accuracy studies
WBC	White blood cell count
